# OnTARi: an ontology for factors influencing therapy adherence to rehabilitation

**DOI:** 10.1186/s12911-021-01512-y

**Published:** 2021-05-11

**Authors:** Bianca Steiner, Birgit Saalfeld, Lena Elgert, Reinhold Haux, Klaus-Hendrik Wolf

**Affiliations:** 1 Peter L. Reichertz Institute for Medical Informatics of TU Braunschweig and Hannover Medical School, Braunschweig, Germany; 2 Peter L. Reichertz Institute for Medical Informatics of TU Braunschweig and Hannover Medical School, Hannover, Germany

**Keywords:** Ontology, Knowledge bases, Knowledge discovery, Treatment Adherence and compliance, Motivation, Health behavior, Psychological theory, Rehabilitation, Chronic disease

## Abstract

**Background:**

Adherence and motivation are key factors for successful treatment of patients with chronic diseases, especially in long-term care processes like rehabilitation. However, only a few patients achieve good treatment adherence. The causes are manifold. Adherence-influencing factors vary depending on indications, therapies, and individuals. Positive and negative effects are rarely confirmed or even contradictory. An ontology seems to be convenient to represent existing knowledge in this domain and to make it available for information retrieval.

**Methods:**

First, a manual data extraction of current knowledge in the domain of treatment adherence in rehabilitation was conducted. Data was retrieved from various sources, including basic literature, scientific publications, and health behavior models. Second, all adherence and motivation factors identified were formalized according to the ontology development methodology METHONTOLOGY. This comprises the specification, conceptualization, formalization, and implementation of the ontology “**On**tology for factors influencing **t**herapy **a**dherence to **r**ehab**i**litation” (OnTARi) in Protégé. A taxonomy-oriented evaluation was conducted by two domain experts.

**Results:**

OnTARi includes 281 classes implemented in ontology web language, ten object properties, 22 data properties, 1440 logical axioms, 244 individuals, and 1023 annotations. Six higher-level classes are differentiated: (1) *Adherence*, (2) *AdherenceFactors*, (3) *AdherenceFactorCategory*, (4) *Rehabilitation*, (5) *RehabilitationForm*, and (6) *RehabilitationType*. By means of the class *AdherenceFactors* 227 adherence factors, thereof 49 hard factors, are represented. Each factor involves a proper description, synonyms, possibly existing acronyms, and a German translation. OnTARi illustrates links between adherence factors through 160 influences-relations. Description logic queries implemented in Protégé allow multiple targeted requests, e.g., for the extraction of adherence factors in a specific rehabilitation area.

**Conclusions:**

With OnTARi, a generic reference model was built to represent potential adherence and motivation factors and their interrelations in rehabilitation of patients with chronic diseases. In terms of information retrieval, this formalization can serve as a basis for implementation and adaptation of conventional rehabilitative measures, taking into account (patient-specific) adherence factors. OnTARi also enables the development of medical assistance systems to increase motivation and adherence in rehabilitation processes.

**Supplementary Information:**

The online version contains supplementary material available at 10.1186/s12911-021-01512-y.

## Background

Rehabilitation, as the third pillar of the German healthcare system, describes transinstitutional, interdisciplinary care processes with the aim of “*[…] enabling disabled persons to reach and maintain their optimal physical, sensory, intellectual, psychological and social functional levels*” [1]. Among the indications for rehabilitation are chronic neurological, cardiological, musculoskeletal, and psychiatric diseases [2]. The therapeutic measures applied are manifold. They range from movement therapy, physical therapy, and pain management to psychological treatment and social counseling to complementary medicine, which includes both naturopathic and alternative medicine treatments [3, 4]. Such therapeutic measures are usually deployed initially for a limited period of time, e.g., over four to six weeks during an full day outpatient or inpatient medical or vocational rehabilitation [3]. For a successful and long-term achievement of therapeutic objectives, however, both a sustainable implementation of behavioral and lifestyle changes practiced in medical or vocational rehabilitation and a long-term provision of subsequent rehabilitation services are crucial [5]. On average, though, only 50% of patients with chronic diseases achieve good treatment adherence [6].

Generally, adherence can be described as „*[…] the extent to which a person’s behavior – taking medication, following a diet, and/or executing lifestyle changes, corresponds with agreed recommendations from a health care provider*” [6]. In rehabilitation, which is not only characterized by individual measures, but passes various phases, it seems advisable to extend this definition with regard to the recommended measures. Accordingly, not only the extent to which a physician’s or therapist's recommendations are followed should be classified as therapy adherence, but rather the adherence to general measures for the implementation of an effective therapy, "*[…] regardless of who recommended it in a specific case*" [7]. Thereby, a multitude of factors exist, which influence the adherence of patients with chronic diseases, either positively or negatively [8]. In 2003, the World Health Organization (WHO) was able to identify 173 different predictors of adherence in nine different indications [6]. Pursuant to their analyzes five categories need to be distinguished: (1) patient-related, (2) social- and economic-related, (3) therapy-related, (4) condition-related, and (5) health system/healthcare team-related factors [6]. It should be noted that the evidence of individual adherence factors varies depending on indications and therapies [8]. Furthermore, concrete effects are rarely proven [9]. Consequently, it is quite difficult to systematically address and overcome such adherence predictors for a specific patient.

Altogether, it becomes clear that there is an enormous number of adherence factors important in rehabilitation processes. These show a high heterogeneity, especially with regard to individual patients. Positive or negative effects on adherence are thus rarely confirmed or even contradicted. Ontologies have been proven in related areas to be convenient to present the existing knowledge on adherence factors in rehabilitation processes from different sources and to make them available for information retrieval, without having to explicitly address the effects of individual adherence factors. For example, the domain ontology OPTImAL successfully formalize predictors that may influence adherence to physical activity and exercise training in the context of rehabilitative treatment of cardiovascular diseases [10]. Ontologies are also frequently used to directly support rehabilitative processes, such as the standard care for rehabilitation of knee conditions [11] or for planning and adapting physiotherapeutic exercises in rehabilitation of musculoskeletal shoulder disorders [12]. However, no ontology seems to exist that provides an overview of possible adherence factors in rehabilitation processes in general, independent of the underlying disease. Therefore, the objective of this research is firstly, to extract the existing knowledge in this domain and secondly, to formalize it in an ontology.

## Methods

### Knowledge extraction

Knowledge extraction was done manually by retrieving data from various sources, including textbooks and scientific publications (*non-ontology knowledge*). Initially, a MEDLINE-search via PubMed was carried out. Thereby, reviews dealing with the collection or analysis of adherence and motivation factors in rehabilitation processes should be identified. The search was based on the search term ‘(Rehabilitation[Title/Abstract] OR Rehab[Title/Abstract]) AND (Motivation[Title/Abstract] OR Adherence[Title/Abstract]) AND (Review[Title])’. A restriction was made via the PubMed-interface with the publication type – ‘Scoping Review’, ‘Systematic Review’, and ‘Meta-Analysis’. Titles and abstracts were screened by two independent reviewers. Full texts were analyzed using a qualitative content analysis based on five inductive categories: (1) Indication/population, (2) area of application, (3) rehabilitation phase, (4) adherence factors, and (5) relations. Single phrases were assigned to these categories and documented in tabular form. Adherence factors and relations clearly recognizable as redundant were removed afterwards.

For a more detailed description of patient-related adherence factors, models and theories of health and motivation research were analyzed. In principle, these models serve to understand, explain, and predict the health behavior of individuals by investigating influencing variables and effectiveness mechanisms [13, 14]. By means of health behavior models, not only further patient-related adherence factors could be collected, but also their interactions could be described. Thereby, motivational models seemed to be of particular relevance [14]. The aim of such models is to identify individual factors, so-called predictors, from which it can be deduced whether a person is willing to change his or her behavior or not [14]. Therefore, the most familiar motivational models were analyzed and modelled as concept maps for formalizing the knowledge contained later on.

Given the variability and low evidence regarding the effects of individual factors on patient adherence and motivation, a pure collection of possible factors was made without considering their actual effects [8, 9].

### Ontology specification

The ontology “**On**tology for factors influencing **t**herapy **a**dherence to **r**ehab**i**litation” (OnTARi) was developed following the ontology development methodology METHONTOLOGY [15]. This method is particularly well suited to construct new ontologies without building upon existing ones. Apart from activities and techniques for ontology management, METHONTOLOGY summarizes separate steps for the developement of an ontology, like “*[…] the specification, the conceptualization, the formalization, the implementation and the maintenance […]*” [15]. A theoretical perspective on ontologies was also considered [16, 17].

OnTARi is intended to represent potential adherence factors and their interaction in rehabilitation processes to address them in conventional interventions or by medical assistance systems. This makes OnTARi beneficial not only for healthcare professionals, e.g., in the preparation of treatment programs, but also for (medical) computer scientists and software developers. For the purposes of information retrieval, OnTARi should be able to answer the following questions:

1. Which adherence factors exist in a certain adherence dimension?
2. Which adherence factors are particularly relevant in rehabilitation processes, that is hard adherence factors?
3. Which hard adherence factors exist in a certain adherence dimension?
4. Which (hard) adherence factors are particularly relevant in a certain rehabilitation field?
5. Which adherence factors are influenced by a certain other adherence factor?

Further details on the ontology specification are summarized in Table 1.

**Table 1:** Characteristics of the domain ontology OnTARi

Characteristic	Description
Purpose	OnTARi serves as a reference model to represent potential adherence factors and their relations in rehabilitation processes
Scope	OnTARi is limited to adherence and motivation factors in rehabilitation processes without addressing the underlying indication or the actual effects of individual factors
Implementation	Web ontology language (OWL) via Protégé 5.5.0
Intended users	1. Healthcare professionals (e.g., physicians, physical therapists) 2. Medical informaticians, software developers
Intended use	1. Implementation or adaptation of conventional rehabilitative measures, taking into account (patient-specific) adherence factors 2. Conceptualization and implementation of medical assistance systems to increase motivation and adherence in rehabilitation processes
Requirements	- Available for reuse (open source software) - Coded using standardized terminology - Based on standard knowledge - Systematic development

### Ontology conceptualization and formalization

In accordance with METHONTOLOGY the conceptualization of OnTARi was based on four successive steps. First, a simple glossary of terms was formed, which contains concepts, individuals, and relations as well as their descriptions. Classes and taxonomies were created in a second step to systematize the identified concepts. Step three dealed with the definition of binary relations. Finally, the dictionary of concepts was created.

#### Glossary of terms

The glossary of terms was derived from the preceding knowledge and term extraction. It consists of the identified adherence and motivation factors as well as associated relations. Single statements were summarized to a term or a short phrase and documented with a proper description, synonyms, type of concepts, possibly existing acronyms, and German translations.

#### Classes and taxonomies

To create OnTARi’s taxonomy, a middle-out approach based on the preceding theoretical analyzes for term extraction was chosen. Classifications from medicine, psychology, and socioeconomics as well as taxonomies of other domain ontologies were used to enable reusability and interoperability of OnTARi (see Table 2). First, a division into three dimensions as main classes was made: *Adherence*, *AdherenceFactors*, and *Rehabilitation.* Thereby, the dimension *Adherence* was defined according to the domain ontology OPTImAL [10]. The taxonomy for *AdherenceFactors* was created successively, in a three-level hierarchy: top-, middle-, and bottom-level. The top-level classification served to represent the five adherence dimensions defined by the WHO [6]. For standardized documentation of patient-related adherence factors, the ‘International Classification of Functioning, Disability and Health’ (ICF) in combination with the ‘Dimensions of Treatment Motivation’ (DTM) was applied [18]. Socioeconomic adherence factors were characterized by the socioeconomic status. According to established socio-cultural theories, this is composed of individual aspects that have been modeled at the middle-level. For classification of condition-related adherence factors, the ‘International Statistical Classification Of Diseases And Related Health Problems, 10th revision’ (ICD-10), the WHO definition of health [19], and the specification of funcional capacity by Patterson et al. [20] were used. From an economic perspective healthcare team/system-related adherence factors can be described as resources. For this reason the so-called ‘Resource-Based View’, a theory recognized among economists, was used for categorization of such factors [21]. As supplement, the perspective of social sciences, i.e. the ‘Taxonomy of Resources’, was included to depict interpersonal resources in addition to economic ones [22].

**Table 2:** Hierarchy of the dimension *AdherenceFactors* with reuse of existing classifications

Top-level	Middle-level	Used classifications
PatientRelatedFactors	DemographicCharacteristics	ICF
	BehavioralFactors	ICF
	HealthAttitudesAndBeliefs	ICF
	PsychologicalFactors	ICF
	Personality	None
	Experiences	DTM
	Knowledge	DTM
	IllnessPerception	DTM
SocioeconomicFactors	SocioeconomicStatus	Theories of social structure
	Education	Theories of social structure
	Employment	Theories of social structure
	WorkSituation	Theories of social structure
	FinancialSituation	Theories of social structure
	FamilialStatus	Theories of social structure
	FamilialResponsibilities	Theories of social structure
	MaritalStatus	Theories of social structure
	HealthInsurance	None
	SocialSupport	ICF
	PersonalAndCommunityResources	None
	CostsOfCare	None
	PhysicalEnvironmentCharacteristics	None
TherapyRelatedFactors	ExerciseRelatedFactors	None
	MedicationRelatedFactors	None
	SurgeryRelatedFactors	None
	PlanningAndImplementationOfTherapyRelatedFactors	None
	GeographicalFactors	None
	AdverseEffectsOfTreatment	None
	AssistiveDevices	None
ConditionRelatedFactors	HealthStatus	WHO
	FunctionalFactors	Patterson et al
	SignsAndSymptomRelatedFactors	None
	Comorbidity	ICD-10
	DiseaseRelatedFactors	None
HealthcareTeamAndSystemRelatedFactors	FinancialFactors	Resource-Based View
	HumanFactors	Resource-Based View
	InterpersonalFactors	Taxonomy of Resources
	SocioculturalFactors	Taxonomy of Resources

The dimension *Rehabilitation* was intended to assign individual adherence factors to a rehabilitation area. This way it should be possible to constitute which adherence factors are of special relevance in a certain rehabilitation area. Thereby, a subdivision into typical areas of rehabilitation, e.g., neurological, internistic, and orthopedic rehabilitation, was done.

#### Definition of binary relations

For the definition of binary relations object and data properties must be differentiated. While object properties represent relations between two classes, data properties can be seen as attributes of a class. Object properties were defined following the principles of object-oriented programming. This means, that as many standardized relations as possible should be implemented, e.g., inheritances and object compositions. Only a few relations should be created specifically for OnTARi. Also data properties should be defined as generically as possible to be applicable in multiple classes.

#### Dictionary of concepts

The dictionary of concepts was mainly built on the adherence predictors identified in literature. Adherence predictors categorized in line with the bottom-up approach used were defined as individuals in OnTARi. For example, the adherence predictor *forgetfulness* is an instance of the class *MemoryAbility*, and the factor *lack of clear instructions from the healthcare professionals* an instance of the class *TrainingAndGuidanceOfPatients*. The dictionary was extended by typical expressions of a class by using existing classifications and taxonomies. LOINC-Codes, for instance, were used to add the individuals *married*, *living in a partnership*, *separated*, *unmarried*, *divorced*, and *widowed* to the class *MaritalStatus*. Individuals initially not defined must be supplemented later on with patient profiles stored in an associated database.

The dictionary also specifies attributes. This includes unique names of attributes, names of concepts to which an attribute is assigned, types, value ranges, and cardinalities. For example, the attribute *has_severity* of the class *Comorbidity* was defined as a string with the value set *extremely mild*, *mild*, *moderate*, *severe*, *extremely severe* and cardinality 1.

### Evaluation and re-design

A taxonomy-oriented evaluation by two domain experts – a medical informatician and a physical therapist – was used to verify OnTARi’s conciseness, consistency, and completeness in terms of classes, object properties, and instances before implementation [23]. Each expert received OnTARi's taxonomy as an Excel spreadsheet and a web ontology language (OWL) file, the associated ontology specification, and an individual evaluation form to document conceptualization errors. Here, eight types of errors in four categories were documented: (1) inconsistency (circularity errors, semantic inconsistency, and overlaps), (2) incompleteness (incomplete concepts and partitioning errors), (3) redundancy (redundant concepts and identical definitions), (4) expression errors (incorrect/unambiguous formulations of concepts). In total, 42 inconsistencies, 43 incompletions, four redundancies and six expression errors could be detected and adjusted accordingly (*re-design*). More details on the expert evaluation can be found in Additional file 1.

### Implementation

The implementation of OnTARi was realized in OWL 2 by using the ontology editor Protégé in version 5.5.0 [24]. Interclass relations were implemented as ‘object restrictions’ using the predefined ‘object properties’. Acronyms, synonyms, definitions, and German translations were embedded via ‘annotation properties’ as subclass of ‘rdfs:comments’ with the datatype ‘rdfs:Literal’.

## Results

### Knowledge base

There is a variety of different health and motivation theories dealing with the analysis and description of behavior and the facilitation of behavioral change. Also in specialist literature, both textbooks and scientific publications, more and more work on therapy adherence and patient motivation can be found, focusing on a wide range of indications and thus care processes.

#### Adherence factors in textbooks

Treatment adherence in rehabilitation research is a fairly new discipline with few concrete research to date [25]. Probably the best known and most comprehensive work stems from the WHO project "Adherence to Long-term Therapies", launched in 2001. Their report "Adherence to Long-term Therapies: Evidence for actions" [6] from 2003, provides a collection of adherence factors as well as a list of possible interventions for individual indications, patients, and settings to increase treatment adherence. Indication-specific adherence factors were identified through individual reviews and assigned to five adherence dimensions: (1) patient-related (n = 51), (2) social- and economic-related (n = 41), (3) therapy-related (n = 27), (4) condition-related (n = 23), and (5) health system/healthcare team-related adherence factors (n = 30). Apart from asthma, cancer, depression, and diabetes, the report also includes reviews on epilepsy, HIV/AIDS, hypertension, tuberculosis, and tobacco control. Altogether 173 different adherence predictors could be determined. Frequently mentioned and therefore easy to generalize factors having a negative impact on adherence include *complex treatments*, *side effects*, *poor working-alliance between healthcare professionals and patients*, *high frequency of treatments or therapeutic sessions*, *mental comorbidities*, *lack of social support and family problems*, *forgetfulness*, and *poor understanding of disease and symptoms*.

#### Adherence factors in scientific publications

Based on the MEDLINE-search described above, 12 reviews on adherence in rehabilitation could be identified [9, 26–36]. The majority deals with the analysis of treatment adherence in terms of cardiovascular diseases (n = 6), especially in acute myocardial infarction [26–31]. Two additional reviews focus on adherence and patient compliance in neurodegenerative diseases [32, 33]. A single one addresses the identification of adherence factors in the outpatient care of cancer [34]. The other three reviews do not have a specific target group [9, 35, 36]. Hall et al. [36] and Essery et al. [9] examine cardiological, neurodegenerative, and musculosceletal diseases together. According to Essery et al. [9], many influencing adherence factors are transferable to other indications or rehabilitation in general (*generalizability*). This applies in particular to adherence factors that could be found in different indications and rehabilitation processes, such as *intention, intrinsic motivation, self-efficacy, previous adherence behavior,* and *social support*.

In total, the analysis revealed 205 different adherence factors. Thereby, the focus is on patient-related factors. Healthcare team and system-related adherence factors are considered only marginally, with *recommendations from healthcare professionals* (n = 3) and *referrals from physicians* (n = 4) being repeated aspects. The most common mentioned socio-economic adherence factors are s*ocial support from family and friends* (n = 8), *access to treatment*, i.e. distance, location, and accessibility of treatment (n = 7), as well as *employment status* (n = 5). Concerning the (general) health status of an individual, adherence factors such as *depression* (n = 7), *smoking* (n = 6), *body mass index* (n = 4), and *physical activity and fitness* (n = 4) can be identified as influences. However, it should be emphasized that individuals with basically good physical, mental, and emotional health are more likely to be adherent than individuals who have comorbidities or feel too ill to participate in treatment. Alongside *age* (n = 6) and *gender* (n = 6), *anxiety and fear* (n = 7) as well as *self-efficacy* (n = 6) and *motivation* (n = 5) are among the most frequently mentioned patient-related adherence factors. Even if the extent of influences on adherence is difficult to determine and varies from individual to individual, it can be stated that *social support by family or friends*, *intention* to carry out therapeutic measures, *intrinsic motivation* and *adherence (history)* to date are among the strongest predictors.

Relations, i.e. dependencies and correlations between adherence factors, are rarely analyzed in literature. Most of the 103 relations determined are based on general statements or assumptions without evidence-based proof.

#### Adherence factors in health and motivation theories

**Fig. 1:**
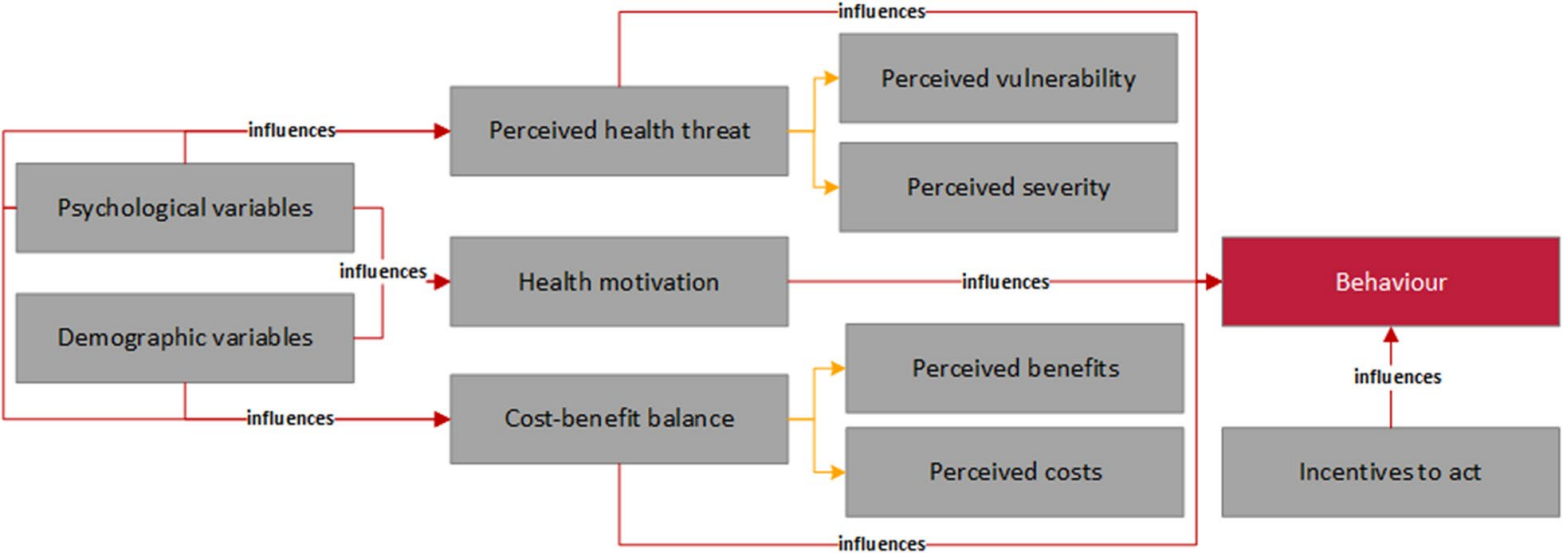
Concept map for the Health Beliefs Model [13]

Motivational models of health behavior assume that positive behavioural changes are all the more likely the more influencing factors are present [14]. One of the first health behavior models is the Health Belief Model (HBM) shown in Fig. 1 [13]. It proceeds from the basic assumption that the probability for a healthy behavior of an individual becomes the more likely, the more this person estimates its *perceived health threat*. The level of personal health threat is estimated to depend on various *demographic and psychological variables*. Likewise a *cost–benefit balance* individually noticed as positively increases the probability for a behavior change. Other factors mentioned in the HBM are *health motivation* and *incentives to act*, such as the opinion of relatives or the severity of self-perceived symptoms. An extension of the HBM is the Protection Motivation Theory (PMT) [13, 37]. Here, *fear appeals* play a central role. Although they do not have a direct effect on a person’s behavior, they address the so-called protection motivation, which is better known as *intention to change a behavior*. Intention depends essentially on two parallel processes, *perceived health threat* and *coping appraisal.* In contrast to the HBM, perceived health threat is not only based on *perceived severity of the disease* and *perceived vulnerability*, but also of *intrinsic and extrinsic rewards*. While health threats increases with perceived vulnerability and severity, they may decrease with higher intrinsic rewards for unhealthy behavior or an already manifested positive experience with such a behavior – ‘I feel better when I am not on a diet’.

Another widely used theory of health behavior is the Theory of Planned Behavior (TPB) [13, 37]. The TPB focuses on the analysis of *competence awareness*. This includes the self-efficacy already known from PMT, here called *perceived behavior control*. An essential assumption is that self-efficacy no longer only has an effect on the intention to act but also directly influences the behavior of an individual. The TPB adds factors influencing self-efficacy, such as *control beliefs* and a person's *subjective strength*. *Attitudes* can have a reinforcing or mitigating effect on intention. For example, attitudes describe either positive or negative ratings of target behaviors – ‘Healthy, vegetarian nutrition is in vogue, […] is only for ecologicals, […] is fun’. A theory very similar to the TPB, but with a stronger focus on social components of behavior, is the Social-Cognitive Theory (SCT) [10]. It assumes that every person who has problems receives, more or less, help from outside. Thus, socio-cultural factors, such as *social support*, also influence the *objectives an individual* (intention) wants to achieve. However, the strongest predictor remains self-efficacy. It depends on one's own *experiences* (strongest predictor), *observational learning*, and *verbal persuasion*.

### Description of OnTARi

**Fig. 2:**
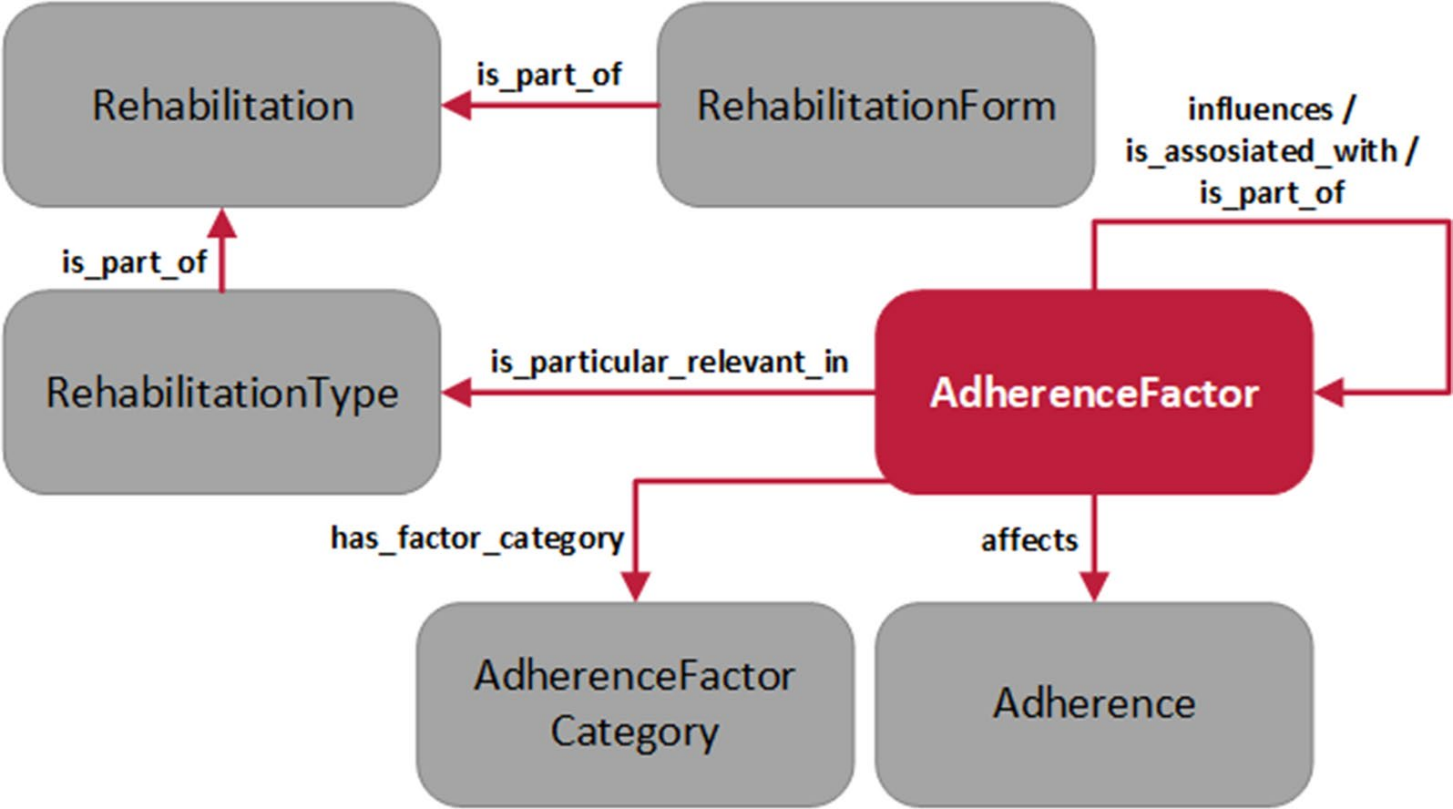
OnTARi-Metamodel

OnTARi includes 281 classes implemented in OWL 2, ten object properties, 22 data properties, 1440 logical axioms, 244 individuals, and 1023 annotations. Thus, 227 different adherence factors are described and assigned to an *AdherenceFactorCategory* (see Fig. 2). Even if the effects of adherence factors differ from individual to individual, a differentiation between *HardFactors* and *SoftFactors* can be made to indicate tendencies. Adherence factors that are particularly likely to have an influence on a person’s adherence are classified as hard factors (n_HardFactor_ = 49), all others as soft factors (n_SoftFactor_ = 178). To represent relations and dependencies among adherence factors, 160 *influences*- and 15 *associated_with*-properties are modeled.

#### Classes and class hierarchy

As seen in OnTARi’s metamodel (Fig. 2), a differentiation between six higher-level classes is made: (1) *Adherence*, (2) *AdherenceFactors*, (3) *AdherenceFactorCategory*, (4) *Rehabilitation*, (5) *RehabilitationForm*, and (6) *RehabilitationType*. According to the analyzed domain ontology OPTImAL, the class *Adherence*, is composed of the level and quality of adherence (*attributes*) [10]. While a person's level of adherence can be measured comparatively clearly, for example by the frequency of performing a therapeutic measure, the quality of adherence refers to an abstract multidimensional construct which is hard to determine.

**Fig. 3:**
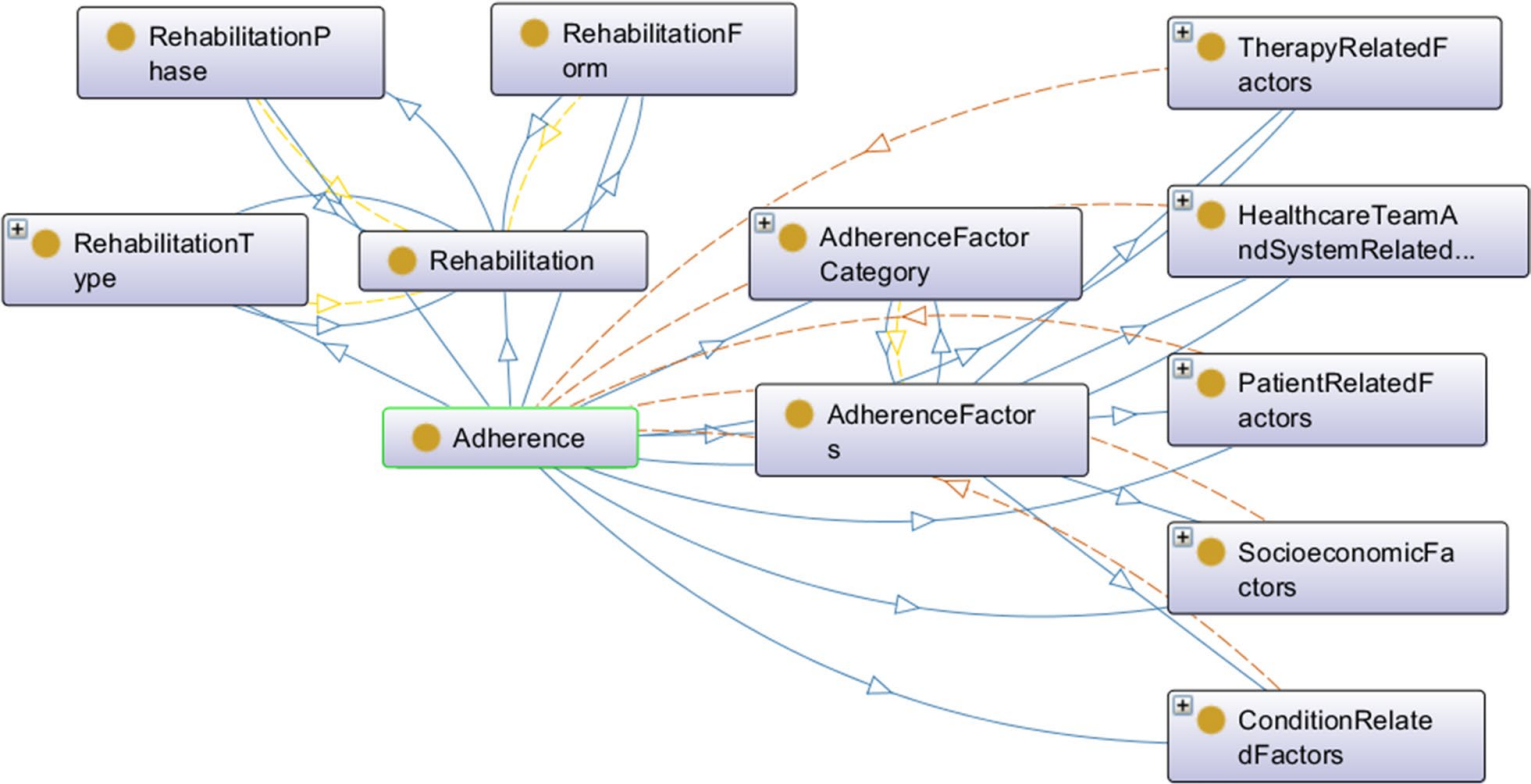
OnTARi’s higher-level classes visualized as OntoGraf

At the top-level of the class *AdherenceFactors* there is a distinctions between the five adherence dimensions defined by the WHO (see Fig. 3). Patient-related adherence factors are classified according to ICF as *DemographicCharacteristics*, *HealthAttitudesAndBeliefs*, *BehaviouralFactors*, and *PsychologicalFactors*. Psychological factors include many aspects known from health behavior models, such as intention, intrinsic and extrinsic motivation, self-efficacy, self-esteem, and coping appraisal. Socioeconomic adherence factors are categorized using individual aspects of a patient’s *SocioeconomicStatus*, including *Education*, *EmploymentStatus*, *WorkSituation*, *FinancialSituation*, *FamilyStructure*, and *MaritalStatus*. Likewise, *SocialSupport* from family, friends, co-workers, and networks, as well as the *TypeOfSupport* – informational, emotional, instrumental – play an essential role here. Therapy-related adherence factors can be classified according to specific therapeutic measures, i.e. exercises, medication, and surgery. Thereby, not only the taste of medications, dosage of medication, and co-prescribings are among the influencing factors, but also the components of exercises, number of exercises to be done, and the extent of surgery. In addition, there are also generic therapy-related factors describing the *PlanningAndImplementationOfTherapy*, such as the target of treatment, required lifestyle changes, complexity of treatment, duration of treatment, and the format of therapy sessions. Condition-related adherence factors essentially include the mental and physical health status of patients as well as a variety of *FunctionalFactors* describing the physical, psychological, and cognitive functioning of patients. But also signs- and symptom-related, comorbidity-related, and primary disease-related factors are relevant. Healthcare team- and system-related factors consists of interpersonal, human, sociocultural, and financial factors. Especially important are the *TherapeuticRelationship*, the *TrustInHealthCareProfessionals*, the *TrainingOfHealthCareProfessionals*, and the *ReferralByPhysicians*.

The class *Rehabilitation* is composed of the *is_part_of* classes *RehabilitationType*, *RehabilitationForm*, and *RehabilitationPhase*. The class *RehabilitationType* contains various subclasses covering typical areas of rehabilitation. This way, it is possible to constitute which factors are of special relevance in a certain rehabilitation area. A subdivision into neurological (n = 84), orthopedic (n = 45), psychosomatic and psychological (n = 9) incl. addiction (n = 15), pediatric, geriatric, gynecological, and internistic rehabilitation is provided. Internistic rehabilitation is once again divided into single sub-classes: cardiological (n = 103), gastroenterological, metabolic (n = 18), oncological (n = 45), and pulmonary (n = 17) rehabilitation. Psychological distress, for example, is a particularly relevant factor in metabolic and oncological rehabilitation.

**Fig. 4:**
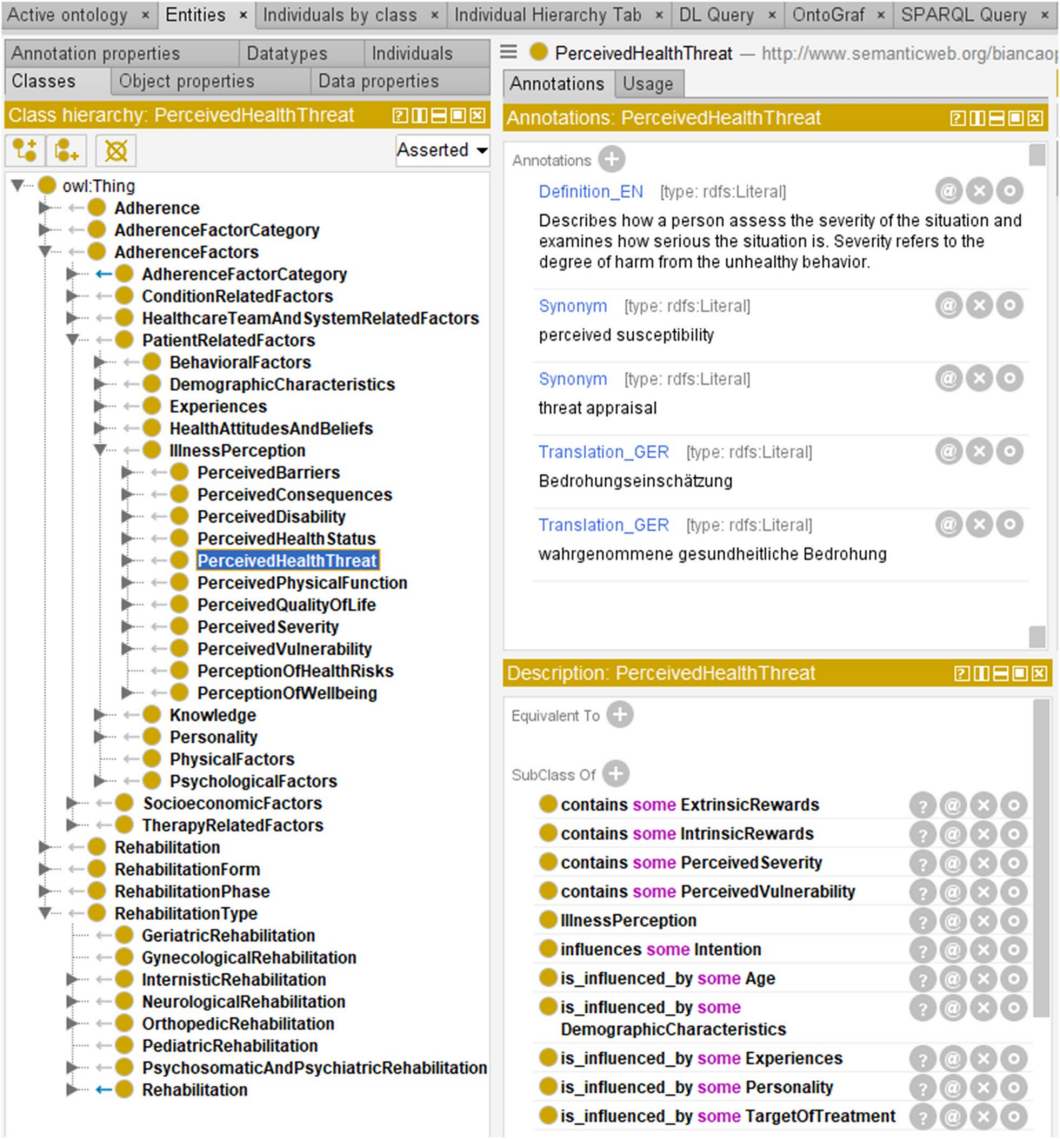
OnTARi excerpt in Protégé

An excerpt of the implemented class hierarchy as well as annotations and relations are shown in Fig. 4.

#### Object properties

**Fig. 5:**
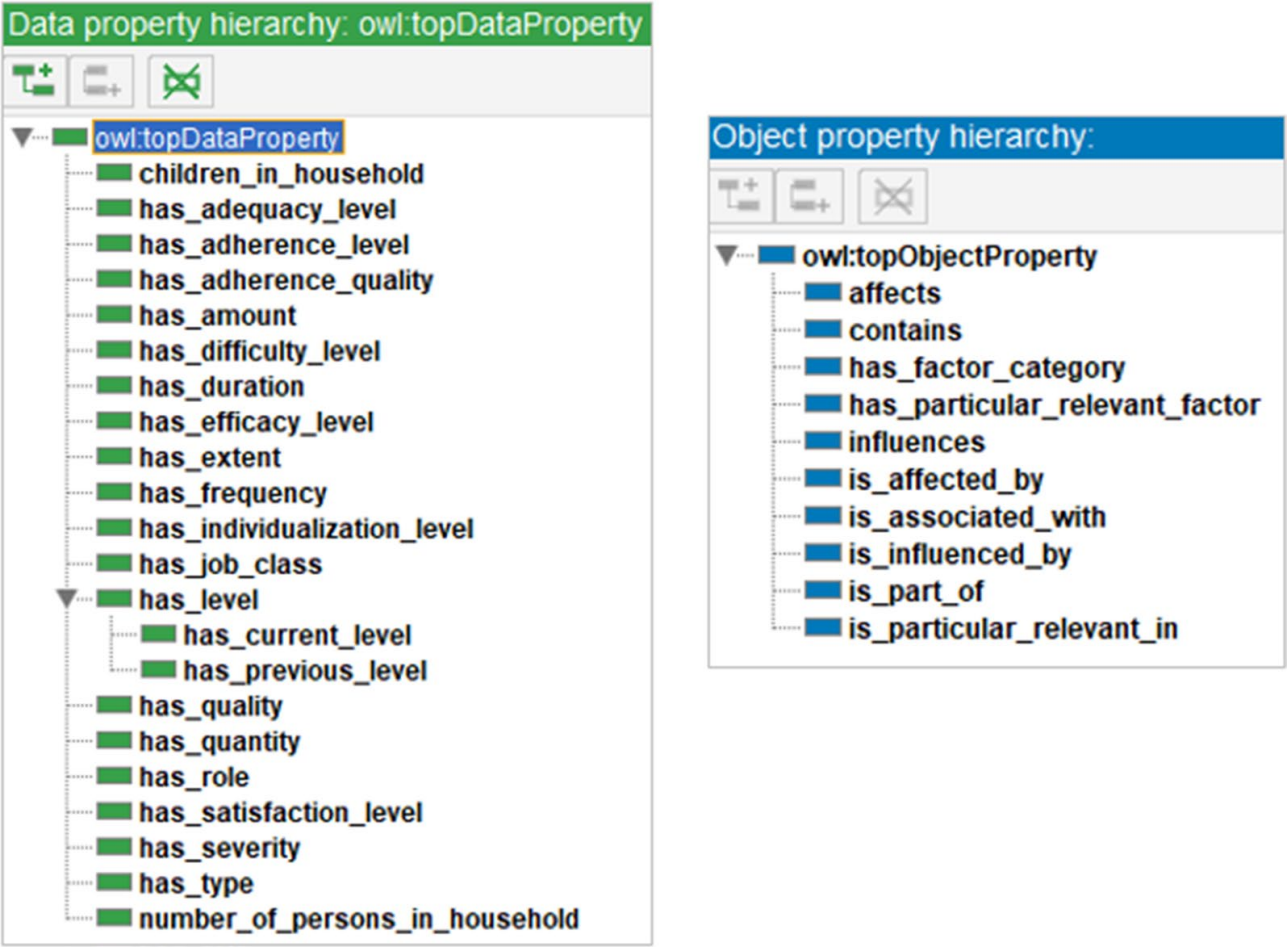
Overview of implemented object and data properties

As seen in Fig. 5, OnTARi defines two standard object properties: *is_a* and *is_part_of*. The *is_a-*relation represents a conventional inheritance between a basic class and the corresponding subclass in the sense of object-oriented programming and knowledge representation. Also the *is_part_of-*relation originates from object-oriented programming, the so-called object compositions. In OnTARi, they are used to model attributes of a class as independent classes without losing the logical structure of information, i.e. the attribute remains an integral part of the state of a class. This procedure is necessary if an attribute represents an adherence factor itself.

Five object properties specially defined for OnTARi are *influences*, *is_associated_with*, *affects*, *has_factor_category* and *is_particular_relevant_in*. The *influences-*relation is used to express that one adherence factor can influence another factor, either positively or negatively. In addition, the *is_associated_with*-relation defines unspecific relationships between adherence factors. For example, there is a correlation between the age and the occurrence of comorbidities. However, this does not mean that the age influences comorbidities, only that the probability of occurrence increases with an older age.

#### Data properties

OnTARi specifies four generic data properties reusable in multiple classes: *has_status*, *has_type*, *has_quality*, and *has_level.* However, such reuse is not always possible. For this reason, there are a number of other individual data properties, such as *has_job_class* for a more detailed description of occupational situations or *children_in_household* for defining family structures. An overview of data properties implemented is shown in Fig. 5.

### Use of OnTARi

According to the objectives defined in advance, OnTARi is intended, among other things, to serve as a basis for the development of medical assistance systems that can address patient-specific adherence factors as precisely as possible. Requests are made directly via Protégé using simple predicate logic, the so-called description logic queries (DL queries). Using the ELK 0.4.3 Reasoner, OnTARi answers questions like those listed in Table 3.

**Table 3:** Generic DL queries used to request OnTARi

Question	Generic DL queries
1	[AdherenceDimension]
2	AdherenceFactors and has_factor_category some HardFactor
3	[AdherenceDimension] and has_factor_category some HardFactor
4.1	AdherenceFactors and is_particular_relevant_in some [RehabilitationType]
4.2	[AdherenceDimension] and has_factor_category some HardFactor and is_particular_relevant_in some [RehabilitationType]
5	[AdherenceFactors] and is_influenced_by some [AdherenceFactor]

**Fig. 6:**
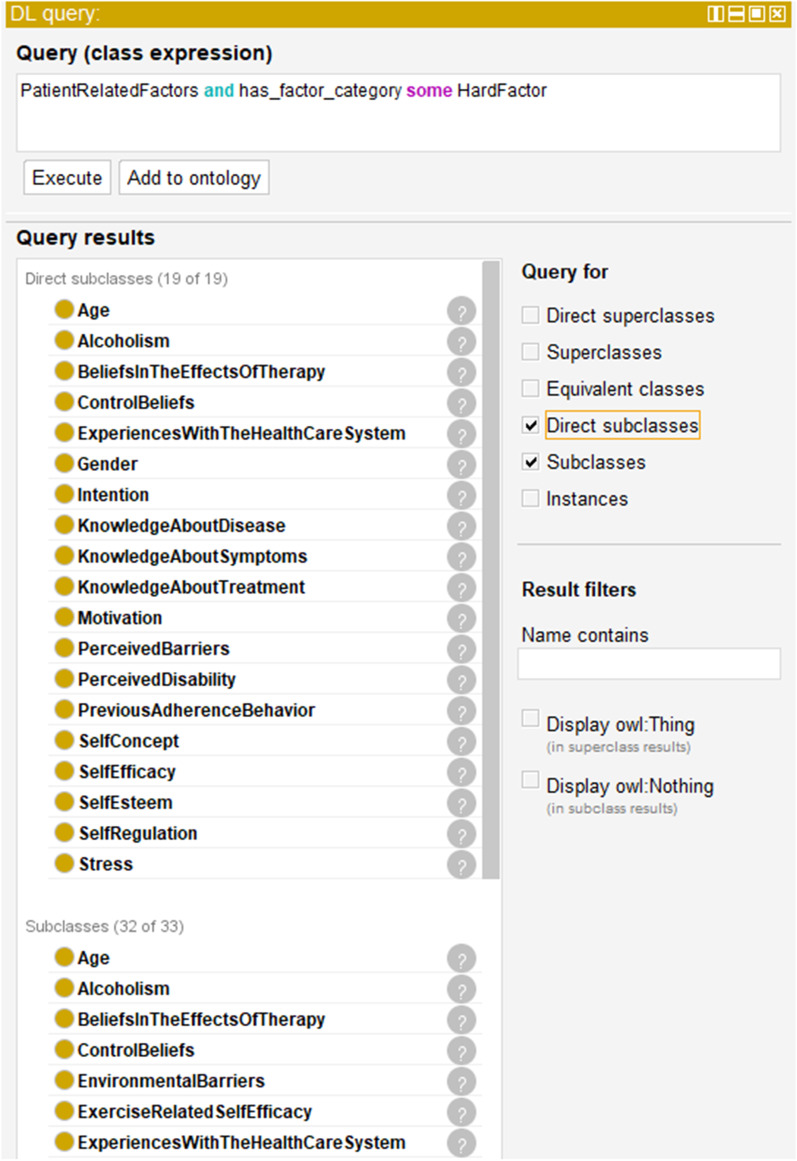
DL query taking hard adherence factors of the dimension patient-related adherence factors

The query ‘PatientRelatedFactors and has_factor_category some HardFactor’, for example, returns all 32 hard factors of the adherence dimension patient-related factors – 19 direct subclasses, 13 indirect subclasses. A part of this query including results is shown in Fig. 6.

## Discussion

Based on a broad data extraction from promising textbooks, established models of health behavior, and systematic reviews the ontology OnTARi was successfully developed according to the method METHONTOLOGY. Implemented in OWL 2 via Protégé, OnTARi includes a total of 281 classes representing 227 different adherence factors. Thereby, a differentiation between hard and soft factors can be made to indicate tendencies of effects. With OnTARi potential adherence factors of individual patients or patient groups can be easily identified via DL queries and finally targeted. For example, it can be observed that the adherence factor *perceived health threat* is influenced, among other things, by the *personality* of an individual. This makes clear that sensitive individuals are more likely to perceive a situation as threatening than individuals with a more self-aware and down-to-earth nature.

In addition to textbooks and theories of health behavior, systematic reviews and meta-analyses were included in the identification of potential adherence factors. There was no separate analysis of studies on adherence and motivation in rehabilitation. During the title and abstract screening in PubMed, it became apparent that there were further isolated studies not covered by the identified reviews. However, it can be assumed that these studies provide few new insights, i.e. additional previously unidentified adherence factors, due to the large database of reviews already available (*data saturation*).

In line with El-Sappagh et al. [38], multiple quality criteria have to be taken into account when developing ontologies. Thus, OnTARi was also systematically developed based on standard knowledge using existing terminologies. However, like many other ontologies, OnTARi is neither based on a consolidated top-level ontology, nor does it take into account inter-ontology interoperability [38, 39]. This hinders the reuse of OnTARi in combination with other ontologies. Also in terms of completeness, OnTARi has weaknesses, especially with regard to the implemented individuals. Whereas completeness was verified for the implemented classes by two independent reviewers, no such evaluation took place for individuals. From the first, only individuals and typical characteristics of a class identified in literature were modeled, e.g., for age, gender, and nationality. However, no claim to completeness was made, as initially only potential adherence factors should be represented. Actual patient profiles are to be added accordingly in future work.

With regard to relations between adherence factors implemented in OnTARi, it can be stated that only relations and dependencies explicitly mentioned in the identified literature were modeled. No supplementation of own or obvious relations between the influencing factors took place to ensure evidence. Yet, at the same time, this reduces the informative value. For example, it is not clear whether an implemented *influences*-relation constitutes a positive or negative effect on therapy adherence. Therefore, it would be conceivable to extend this relation in future work by the sub-relations *increases* and *decreases*, even though they are harder to model. If the effects of an adherence factor are known, e.g., can be derived from scientific theories and studies, a differentiation of predictors and promoting factors in rehabilitation process on the level of individuals would be possible.

Given its generic nature, OnTARi currently only allows implicit mapping of patient profiles. Obviously, the more information is available about a patient, especially about patient-related adherence factors, the more specific the patient profile can be elaborated and the more targeted the identified adherence factors can be addressed. Conversely, this also implies that a minimal set of information about a patient must be available to enable patient-specific queries. This includes demographic information, such as age and gender, indication-specific information, such as diagnosis, duration of therapy and previously perceived measures, as well as the current adherence and motivation levels.

Information retrieval is currently only possible via DL queries. The use of these simple predicate logical expressions makes it possible to quickly and easily obtain an overview of potential adherence factors, especially hard adherence factors in specific rehabilitation areas. More powerful query languages, such as the Protocol And Resource Description Framework Query Language (SPARQL), also offer the possibility of making queries taking into account the individual patient profile. However, using such queries in Protégé is exceedingly complex, particularly for non-computer scientists. Hence, in future work, a suitable graphical user interface should be implemented to allow easier access to OnTARi. By means of this user interface, patient data describing the patient profile could be documented step by step and easily queried.

## Conclusion

A multitude of factors may influence treatment adherence of patients with chronic diseases in rehabilitation, either positively or negatively. The effects, if any, of such factors always depend on the individual patient and are therefore rarely evidenced. The developed ontology OnTARi serves as a generic reference model providing a comprehensive overview of potential adherence and motivation factors and their interrelations in rehabilitation of patients with chronic diseases. Based on the literature review conducted, single adherence factors can be assigned to typical rehabilitation areas, such as cardiological, neurological, or othopedic rehabilitation. This formalization can serve as a basis for implementation and adaptation of conventional rehabilitative measures, taking into account (patient-specific) adherence factors. In addition to direct use as a knowledge base in Protégé, OnTARi can also be used as an information retrieval system or even as a knowledge manager in medical assistance systems to increase motivation and adherence in rehabilitation processes.

## Supplementary Information

**Additional file 1.** Results of the expert evaluation.

## Data Availability

The datasets generated during this work are available in the GitHub repository OnTARi, https://github.com/PLRI/OnTARi. Here the OWL file of the ontology is accessible.

## Abbreviations

DL queries: Description Logic Queries
DTM: Dimensions of Treatment Motivation
HBM: Health Belief Model
ICD-10: International Statistical Classification of Diseases And Related Health Problems, 10th revision
ICF: International Classification of Functioning, Disability and Health
OnTARi: Ontology for factors influencing therapy adherence to rehabilitation
OWL: Ontology Web Language
PMT: Protection Motivation Theory
SPARQL: Protocol And Resource Description Framework Query Language
SCT: Social-Cognitive Theory
TPB: Theory of Planned Behavior
WHO: World Health Organization
